# Genome-wide association study identifies loci and candidate genes for grain micronutrients and quality traits in wheat (*Triticum aestivum* L.)

**DOI:** 10.1038/s41598-022-10618-w

**Published:** 2022-04-29

**Authors:** Nagenahalli Dharmegowda Rathan, Hari Krishna, Ranjith Kumar Ellur, Deepmala Sehgal, Velu Govindan, Arvind Kumar Ahlawat, Gopalareddy Krishnappa, Jai Prakash Jaiswal, Jang Bahadur Singh, Saiprasad SV, Divya Ambati, Sumit Kumar Singh, Kriti Bajpai, Anju Mahendru-Singh

**Affiliations:** 1 Indian Agricultural Research Institute, New Delhi, India; 2 International Maize and Wheat Improvement Center, Texcoco, Mexico; 3 Indian Institute of Wheat and Barley Research, Karnal, India; 4 Govind Ballabh Pant University of Agriculture and Technology, Pantnagar, India; 5 Indian Agricultural Research Institute, Regional Station, Indore, India

**Keywords:** Plant breeding, Heritable quantitative trait, Plant breeding

## Abstract

Malnutrition due to micronutrients and protein deficiency is recognized among the major global health issues. Genetic biofortification of wheat is a cost-effective and sustainable strategy to mitigate the global micronutrient and protein malnutrition. Genomic regions governing grain zinc concentration (GZnC), grain iron concentration (GFeC), grain protein content (GPC), test weight (TW), and thousand kernel weight (TKW) were investigated in a set of 184 diverse bread wheat genotypes through genome-wide association study (GWAS). The GWAS panel was genotyped using Breeders' 35 K Axiom Array and phenotyped in three different environments during 2019–2020. A total of 55 marker-trait associations (MTAs) were identified representing all three sub-genomes of wheat. The highest number of MTAs were identified for GPC (23), followed by TKW (15), TW (11), GFeC (4), and GZnC (2). Further, a stable SNP was identified for TKW, and also pleiotropic regions were identified for GPC and TKW. In silico analysis revealed important putative candidate genes underlying the identified genomic regions such as *F-box-like domain superfamily, Zinc finger CCCH-type proteins, Serine-threonine/tyrosine-protein kinase, Histone deacetylase domain superfamily,* and *SANT/Myb domain superfamily proteins,* etc. The identified novel MTAs will be validated to estimate their effects in different genetic backgrounds for subsequent use in marker-assisted selection.

## Introduction

Micronutrient deficiency, also known as ‘hidden hunger’ is mainly caused by the intake of diets often dominated by food staples low in minerals and vitamins^1^. It affects around two billion people worldwide and causes about 45% of deaths annually of children below five years of age^2^. Around 155 million children suffer from stunting and 52 million are wasted particularly in Asia and Africa^3^. The iron (Fe) and zinc (Zn) deficiencies among the minerals caused due to reduced dietary intake are a greater risk factor for human health^4–6^ and affect about one-third of the population in developing countries^7,8^. The Fe deficiency is indicated by reduced haemoglobin content resulting in anaemia and affects over 24.8% of the population worldwide and about 65% of the preschool-aged children in South-East Asia and Africa^9^. It can lead to several life-threatening diseases such as chronic kidney and heart failure, as well as inflammatory bowel diseases^10^. The Zn deficiency affects 17.3% of the global population mostly in developing countries of Asia and Africa^11^ and is responsible for the death of over half a million children below the age of five years^12^. It induces a wide range of physiological problems, such as growth retardation, impaired brain development, increased vulnerability to infectious diseases, diarrhoea and pneumonia, as well as an increased risk of infant mortality, pregnancy, and childbirth complications, and a range of other chronic diseases^13–15^. The GPC along with nutritional importance also determines the processing and end-product quality of wheat. One of the most common causes of infection in humans is a lack of secondary immunity caused by protein-energy malnutrition (PEM). Marasmus (chronic wasting) or kwashiorkor (edema and anemia) are the two clinical symptoms of acute PEM in infants^16^. In children with chronic PEM, cognitive growth is hampered^17^. In developing countries, micronutrients and protein-energy malnutrition are the leading causes of death, with pregnant women and young children being the most vulnerable^18^.

The cereals contribute to the largest daily dietary intake in the regions where micronutrient deficiencies are most prevalent^5,19^. The staple grains such as wheat (*Triticum aestivum*) and rice (*Oryza sativa*) contain sub-optimal levels of micronutrients especially Fe and Zn and milling further reduces their content. Genetic biofortification of staple food crops through conventional, molecular, or transgenic methods is considered a sustainable cost-effective long-term strategy for addressing nutritional deficiency^20^. The global wheat production for 2020–21 was 772.6 million metric tons (MMT), which is 117 MMT higher than the 2012–13 production of 655 MMT and hence is sufficient to meet global consumption demand^21^. Wheat production in India hit a new high of 109.5 million tonnes in the crop year 2020–21^21^. Wheat is consumed by 2.5 billion people worldwide^22^ and staple food for 30% of the population, particularly in developing countries^23^. It also accounts for one-fifth of the daily caloric intake and provides more than 20% of global dietary energy^24^. Wheat has greater accessibility, adaptability, and increased production to fulfil consumer demand. Therefore, biofortification of wheat in developing countries is expected to effectively reduce micronutrient malnutrition.

Identification of closely linked molecular markers to the genomic regions governing complex quantitative traits like Fe, Zn, and GPC will help in the development of biofortified wheat varieties through marker-aided breeding. Currently, GWAS is the most popular approach for dissecting the genetic basis of complex traits^25,26^. The increased QTL resolution, allele coverage, and ability to use large sets of natural germplasm resources such as landraces, elite cultivars, and advanced breeding lines are advantages of GWAS over conventional QTL mapping based on bi-parental populations. However, GWAS has only been used in a few studies to investigate the genetics of GFeC and GZnC in wheat^27–33^.

The yield penalty and concentration effects are additional bottlenecks for wheat biofortification^34^. Knowledge of the genetic relation of grain micronutrients along with TKW and TW may provide insights for the improvement of micronutrient concentration without compromising grain quality and yield potential. Therefore, more studies are needed on GWAS and also on the identification of candidate genes as well as genomic regions that regulate the accumulation of grain micronutrients and protein concentration. The identified novel genomic regions may be introgressed to develop high-yielding biofortified cultivars. The present study was aimed to identify the genomic region(s) and candidate genes associated with grain Zn and Fe concentration, GPC, TW, and TKW through GWAS in a set of 184 diverse bread wheat genotypes.

## Materials and methods

### Planting material and conduct of experiment

A set of 184 diverse bread wheat genotypes consisting of old and new Indian elite varieties, exotic lines, landraces, synthetic hexaploid, and derived lines were used for GWAS (Supplementary Table S1). The GWAS panel was evaluated for GZnC, GFeC, GPC, TKW, and TW during 2019–20 crop season at three diverse locations viz., IARI-New Delhi (E1) (Indian Agricultural Research Institute, Research Farm, New Delhi located 29.7008° N, 76.9839° E, 228.6 m AMSL), IARI-Indore (E2) (Indian Agricultural Research Institute, Regional Station, Indore located 22.7196° N, 75.8577° E, 228.6 m AMSL) and GBPUAT-Pantnagar (E3) (Govind Ballabh Pant University of Agriculture and Technology, Research Farm, Uttarakhand, 29.0229° N, 79.4879° E, 243.8 m AMSL). Each genotype was grown in a 5 rows plot of 2.5 m each, with a row-to-row distance of 0.25 m following augmented design with repeated checks namely, HD 3086, C 306, HI 1544, and GW 322. The pests and diseases were controlled chemically, whereas weeds were controlled both manually and chemically. Plant materials were harvested after the grains reached physiological maturity and were completely dry in the field.

### Phenotyping

Twenty spikes of each entry were manually harvested, threshed, and carefully cleaned by discarding broken grains and foreign material without touching to metal parts of the farm equipment and used for micronutrient analysis. GZnC and GFeC were measured with a “bench-top” non-destructive, energy-dispersive X-ray fluorescence spectrometry (ED-XRF) instrument (model X-Supreme 8000; Oxford Instruments plc, Abingdon, United Kingdom) standardized for high-throughput screening of mineral concentration of whole-grain wheat^35^. The GZnC and GFeC were expressed in milligrams per kilogram (mg/kg). The GPC was measured by Infra-red transmittance-based instrument Infra-tec 1125 and expressed in percentage (%). The TKW was measured by weighing a set of randomly selected 1000 grains representing the whole grain sample in the Numigral grain counter. To record the TW, a thoroughly cleaned grain sample was poured into the metallic funnel of the hectoliter weight instrument developed by the ICAR-Indian Institute of Wheat and Barley Research, Karnal. After the grain was levelled well, the outlet was opened to allow the free flow of the grain in the metallic tubular container below till it is filled. Then the shutter was slid to remove the excess grain and to level it. The grain contained in the measuring can was then weighed using an electronic balance. The TKW was expressed in grams (g) and TW as kilogram per hectoliter (kg/hl).

### Phenotypic data analyses

The phenotypic data was analysed with ACBD-R (Augmented Complete Block Design with R) version 4.0 software^36^. The mean, coefficient of variation (CV), least significant difference (LSD), genotypic variance, and heritability were estimated. In ACBD-R v4.0, the best linear unbiased predictors (BLUPs) of each genotype were calculated for each environment and across environments along with four checks varieties (HD 3086, C 306, HI 1544, and GW 322). The calculated BLUPs were then used in the GWAS analysis. The frequency distribution graphs and correlation coefficients of the recorded traits were obtained through Past 3.01 software^37^.

### Genotyping

The CTAB method by Murray and Thompson^38^ was used to extract genomic DNA from the leaves of 21-day old seedlings. The genotyping was done using the 35 K Axiom® Wheat Breeder’s Array^39^ by outsourcing to Imperial life sciences, India. A total of 35,153 single nucleotide polymorphism (SNP) markers were processed to obtain high-quality informative markers. The filtering was done in MS Excel and markers with minor allele frequency (MAF) less than 0.05 and greater than 0.95, missing data greater than 30%, and heterozygosity greater than 20% were removed. The remaining set of 9503 high-quality SNP markers was used in GWAS analysis.

### Linkage disequilibrium (LD), population structure, and GWAS

The pairwise LD values (*r*^2^) were estimated in TASSEL version 5.2.79 and the values were plotted against genetic distance (bp) in R Studio following^40^. The pattern of LD decay was determined as the distance where LD values reduced to half of their maximum value.

Population structure was inferred by two independent methods: Principal Component Analysis (PCA) using GAPIT version 3.0^41^, and by neighbour–joining (N-J) clustering method in TASSEL version 5.2.79. For cluster analysis, the distance matrix was generated to construct tree using TASSEL version 5.2.79 software by following N-J clustering method, then the tree file was exported in Newick format to construct N-J tree in iTOL version 6.5.2 (https://itol.embl.de/).

The BLUPs from the 184 genotypes were used as phenotypic data in GWAS along with corresponding genotyping data. Significant marker-trait associations (MTAs) were identified using the Fixed and random model Circulating Probability Unification (FarmCPU) model approach in GAPIT version 3.03^41,42^. This algorithm selects the associated markers as a cofactor to control false positives using likelihood in MLM to avoid overfitting tests markers iteratively. The suitability of the model to account for population structure was assessed using quantile–quantile (Q–Q) plots. SNPs with *p* ≤ 0.0001 were considered significantly associated with individual traits. The past 3.01 software was used to draw box plots to show the allelic effects of the significant MTAs of GZnC and GFeC.

### In silico analysis

A 100 bp sequence was extracted from Ensemble Plants database (http://plants.ensembl.org/index.html) of the bread wheat genome (IWGSC (RefSeq v1.0)) and added on both sides of the SNP for in silico analysis. Insilico search for the putative candidate genes was then done using Basic Local Alignment Search Tool (BLAST) in the Ensemble plant database (https://plants.ensembl.org/index.html). The genes found in the overlapping region and within 1 Mb upstream and downstream of the matched regions were selected as candidate genes and their molecular functions were determined. In addition, their expression patterns were investigated using the Wheat Expression database (http://www.wheat-expression.com/) and potential links to phenotypes were determined using Knetminer tool integrated with Wheat Expression database. The role of the identified putative candidate genes in the regulation of GZnC and GFeC was also determined with the previous reports.

## Results

### Phenotypic evaluations

**Figure 1:**
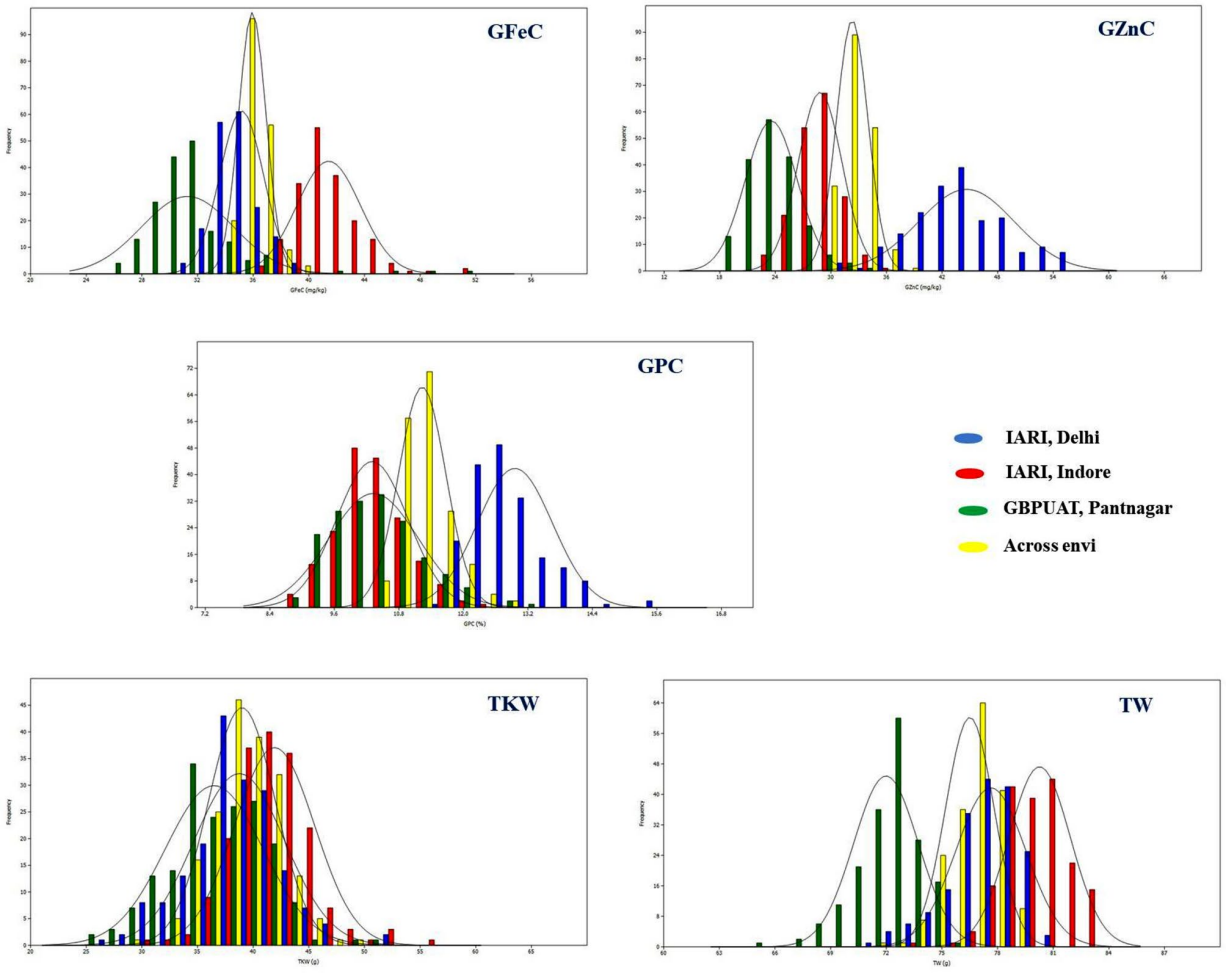
Frequency distribution of GZnC, GFeC, GPC, TKW and TW in the GWAS panel evaluated in IARI-New Delhi (E1), IARI-Indore (E2), GBPUAT-Pantnagar (E3), and across environments (E4).

A set of 184 diverse genotypes in the GWAS panel were evaluated for nutritional and other grain quality traits in three diverse locations viz., IARI-New Delhi (E1), IARI-Indore (E2), GBPUAT-Pantnagar (E3), and the combined data across three environments (E4). The summary statistics including mean, range, coefficient of variance, heritability, and variance estimates are presented in Table 1. The genotypic variance is significant for all the studied traits. An extensive range of variation was observed for all the traits in all the studied environments (Table 1; Fig. 1). The variation for GZnC, GFeC, GPC, TKW, and TW ranged from 17.56 mg/kg to 56.93 mg/kg, 25.47 mg/kg to 52.09 mg/kg, 8.6% to 15.81%, 24.33 g to 57.18 g and 64.48 kg/hl to 83.77 kg/hl, respectively. The heritability estimates for GZnC, GFeC, and GPC were variable and ranged between 0.5–0.88, 0.4–0.8, and 0.56–0.82, respectively, heritability for TKW and TW were greater and ranged between 0.73 and 0.92. Based on the combined BLUP values over the environments, the ten best-performing lines for the traits were selected and presented in Table 2. The landrace Navrattan and Syntheic Hexaploid Wheat (SHW) 2.38 were among the best performing genotypes for GZnC, GFeC, and GPC. The Indian variety Lokbold was found among the best performing lines for GZnC, GFeC, and TKW.

**Table 1:** Genetic parameters from the GWAS panel evaluated in IARI-New Delhi (E1), IARI-Indore (E2), GBPUAT-Pantnagar (E3), and across environments (E4).

Trait	Env	GWAS panel					
		Range	Mean ± SD	CV (%)	LSD	*h*^*2*^	Genotype variance
GZnC (mg/kg)	E1	31.86–56.93	44.64 ± 5.16	11.55	5.58	0.88	30.78***
	E2	22.31–36.91	28.83 ± 2.37	8.22	3.86	0.75	7.61***
	E3	17.56–32.90	23.56 ± 2.80	11.90	4.95	0.72	11.16***
	E4	28.65–37.72	32.30 ± 1.70	5.26	4.73	0.50	5.77***
GFeC (mg/kg)	E1	31.87–39.74	35.17 ± 1.58	4.48	4.21	0.53	4.81*
	E2	36.71–51.38	41.42 ± 2.29	5.53	4.85	0.64	8.37**
	E3	25.47–52.09	31.28 ± 3.31	10.59	4.71	0.80	13.96***
	E4	34.12–40.05	35.94 ± 0.99	2.76	3.45	0.40	2.53***
GPC (%)	E1	11.56–15.81	12.96 ± 0.70	5.39	1.14	0.75	0.66***
	E2	8.77–12.39	10.31 ± 0.67	6.46	0.90	0.82	0.55***
	E3	8.63–13.24	10.33 ± 0.83	8.07	1.31	0.77	0.92***
	E4	10.38–13.00	11.23 ± 0.44	3.93	1.09	0.56	0.35***
TKW (g)	E1	27.79–52.69	38.75 ± 4.09	10.56	3.52	0.92	18.39***
	E2	31.23–57.18	41.92 ± 3.59	8.57	4.04	0.87	15.12***
	E3	24.33–51.53	36.49 ± 4.38	11.99	6.11	0.81	24.20***
	E4	29.54–49.80	38.99 ± 3.01	7.71	4.83	0.75	12.05***
TW (kg/hl)	E1	71.32–81.45	77.64 ± 1.88	2.43	2.61	0.81	4.47***
	E2	74.10–83.77	80.26 ± 1.66	2.07	1.42	0.92	3.04***
	E3	64.48–75.43	71.99 ± 1.74	2.42	3.01	0.73	4.26***
	E4	71.76–79.05	76.50 ± 1.31	1.71	2.22	0.73	2.34***

E1, IARI, New Delhi; E2, IARI, Indore; E3, GBPUAT, Pantnagar; E4, across environments; SD, standard deviation; CV, coefficient of variance; LSD, least significant difference; h^2^, heritability.

*Significant at p < 0.05.

**Significant at p < 0.01.

***Significant at p < 0.001.

**Table 2:** The highest-performing 10 lines for GFeC, GZnC, GPC, TW and TKW in the GWAS panel based on combined BLUPs across environments.

Genotypes	GFeC (mg/kg)	Genotypes	GZnC (mg/kg)	Genotypes	GPC (%)	Genotypes	TW (g)	Genotypes	TKW (g)
Kundan	40.05	SHW (2.38)	37.72	Local collection 1c01	13.00	C273	79.05	Lokbold	49.8
UP2672	39.62	C591	36.41	IITR26	12.67	HD3118	78.93	DL1532	47.23
NW1014	39.36	NP852	36.06	NP852	12.50	HD3354	78.81	Kundan	46.2
SHW (2.38)	38.27	Navrattan	36.05	Navrattan	12.32	HD2982	78.81	CS5	45.94
Hindi62	38.09	QBP128	35.95	VL829	12.29	HI617	78.69	Asocmap260	44.83
Westonia	38.04	Kharchia65	35.88	SHW (2.38)	12.22	HI1563	78.69	UP2425	44.65
Navrattan	37.87	C273	35.63	NP770	12.11	RAJ4120	78.69	DBW187	44.52
HD2932	37.81	NP770	35.46	DBW14	12.00	K68	78.56	PBW752	44.24
Lokbold	37.76	Lokbold	35.26	HD2189	11.99	QBP1210	78.56	PBW689	43.91
HD2189	37.74	K68	34.98	HI1605	11.98	K1317	78.56	DBW43	43.51

**Figure 2:**
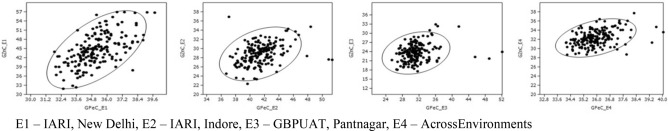
Scatter plots showing the correlation of grain zinc (GZnC) and iron (GFeC) concentration in GWAS panel evaluated in IARI-New Delhi (E1), IARI-Indore (E2), GBPUAT-Pantnagar (E3), and across environments (E4).

The Pearson correlation coefficients (*r*) estimated between the traits in each environment are presented in Table 3. The association was positive and highly significant (*p* < 0.01 to 0.001) among GZnC, GFeC (Fig. 2), and GPC in each environment and across environments. The GFeC was found to have a consistently significant positive correlation with TW and TKW (*p* < 0.05–0.01) in E1, E2, and E4, however, GZnC did not show any correlation with either TW or TKW in any of the environments. Further, the association of GPC with TW and TKW was negative and significant in E2, E3, and E4 (*p* < 0.05–0.01). The TKW showed a strong positive correlation with TW in all the environments (*p* < 0.01–0.001).

**Table 3:** Pair-wise correlation coefficients among the traits in GWAS panel evaluated in IARI-New Delhi (E1), IARI-Indore (E2), GBPUAT-Pantnagar (E3), and across environments (E4).

	Traits	GZnC	GPC	TW	TKW
IARI-New Delhi (E1)	GFeC	0.60***	0.28***	0.18*	0.20**
	GZnC		0.46***	− 0.03	0.01
	GPC			0.03	0.03
	TW				0.50***
IARI-Indore (E2)	GFeC	0.37***	0.22**	0.12	0.17*
	GZnC		0.30***	0.09	− 0.01
	GPC			− 0.19**	− 0.23**
	TW				0.23**
GBPUAT-Pantnagar (E3)	GFeC	0.25***	0.23**	0.06	0
	GZnC		− 0.14	0.1	− 0.04
	GPC			− 0.16*	− 0.15*
	TW				0.50***
Across Env (E4)	GFeC	0.41***	0.39***	0.23**	0.15*
	GZnC		0.30***	0.14	− 0.04
	GPC			0	− 0.18*
	TW				0.34***

*Significant at p < 0.05.

**Significant at p < 0.01.

***Significant at p < 0.001.

### Marker distribution, LD decay and population structure

A set of 9,503 high quality SNP markers were distributed across the genome with the highest number of markers on the B sub-genome (3646), followed by A (2979) and D (2878) sub-genomes, respectively. Chromosome-wise distribution suggests that the highest number of markers were mapped on chromosome 1B (675) followed by chromosome 2B (653) and 1D (610). Chromosomes 4D (170) and 4B (266) had the least number of markers (Table 4).

**Table 4:** The sub-genome-wise distribution of SNP markers in the GWAS panel.

	GWAS panel							
Genome↓/chromosome →	1	2	3	4	5	6	7	Total
A	496	498	402	315	466	336	466	2979
B	675	653	478	266	588	540	446	3646
D	610	597	391	170	436	320	354	2878

**Figure 3:**
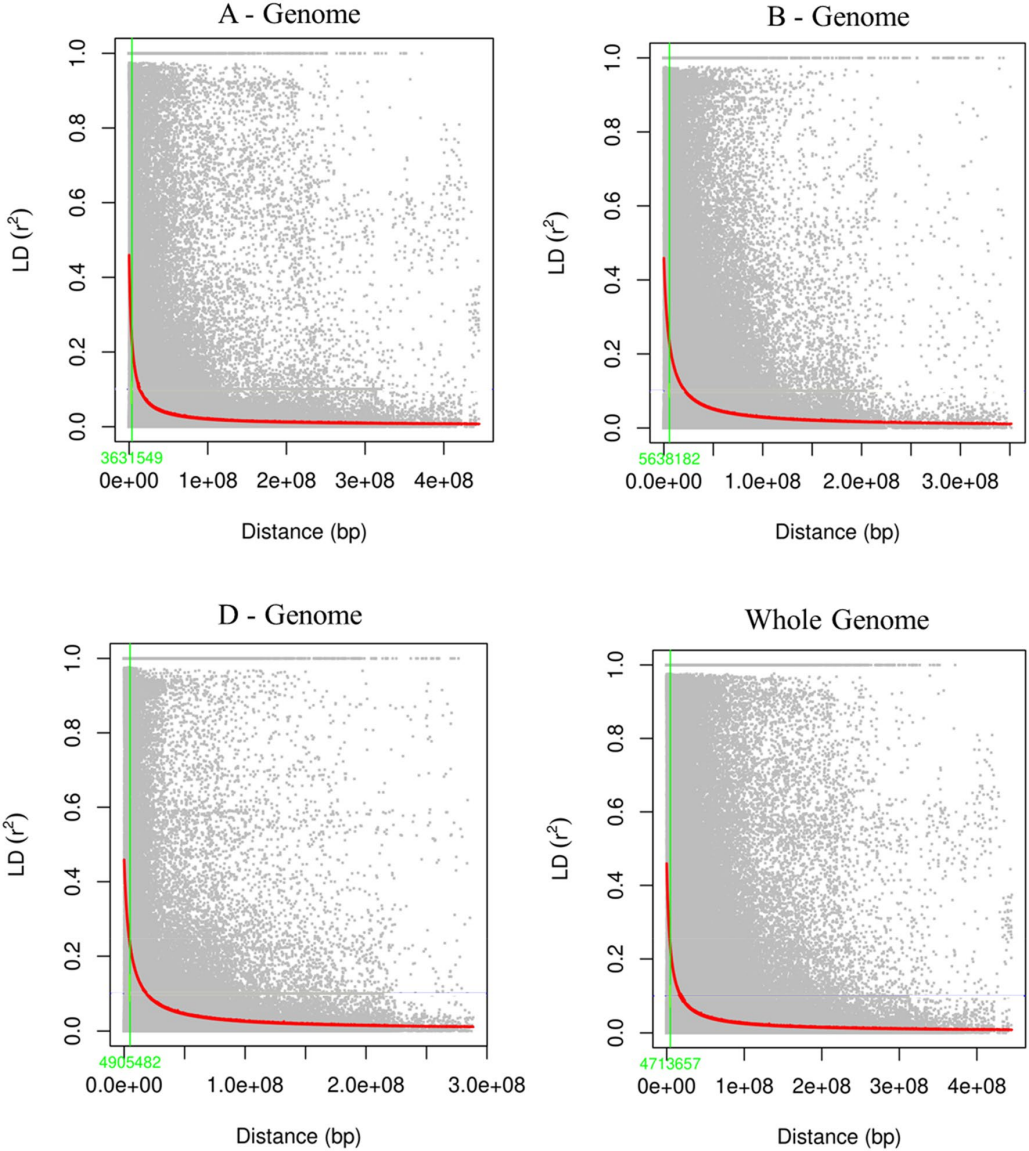
Scatterplot showing linkage disequilibrium (LD) decay estimated by plotting (r^2^) against genetic distance (bp) in 184 diverse bread wheat accessions. The green line indicates the threshold point where LD dropped to 50% of its maximum value. LD decay value is at cut off point is indicated on the x-axis with the green font.

The LD was estimated by calculating the squared correlation coefficient (*r*^*2*^) for all the 9503 markers. Genome-wide LD decayed with genetic distance, the LD decayed to its half at 4.71 Mb for whole genome, and 3.63 Mb for A, 5.63 Mb for B and 4.90 Mb for D sub-genomes (Fig. 3).

**Figure 4:**
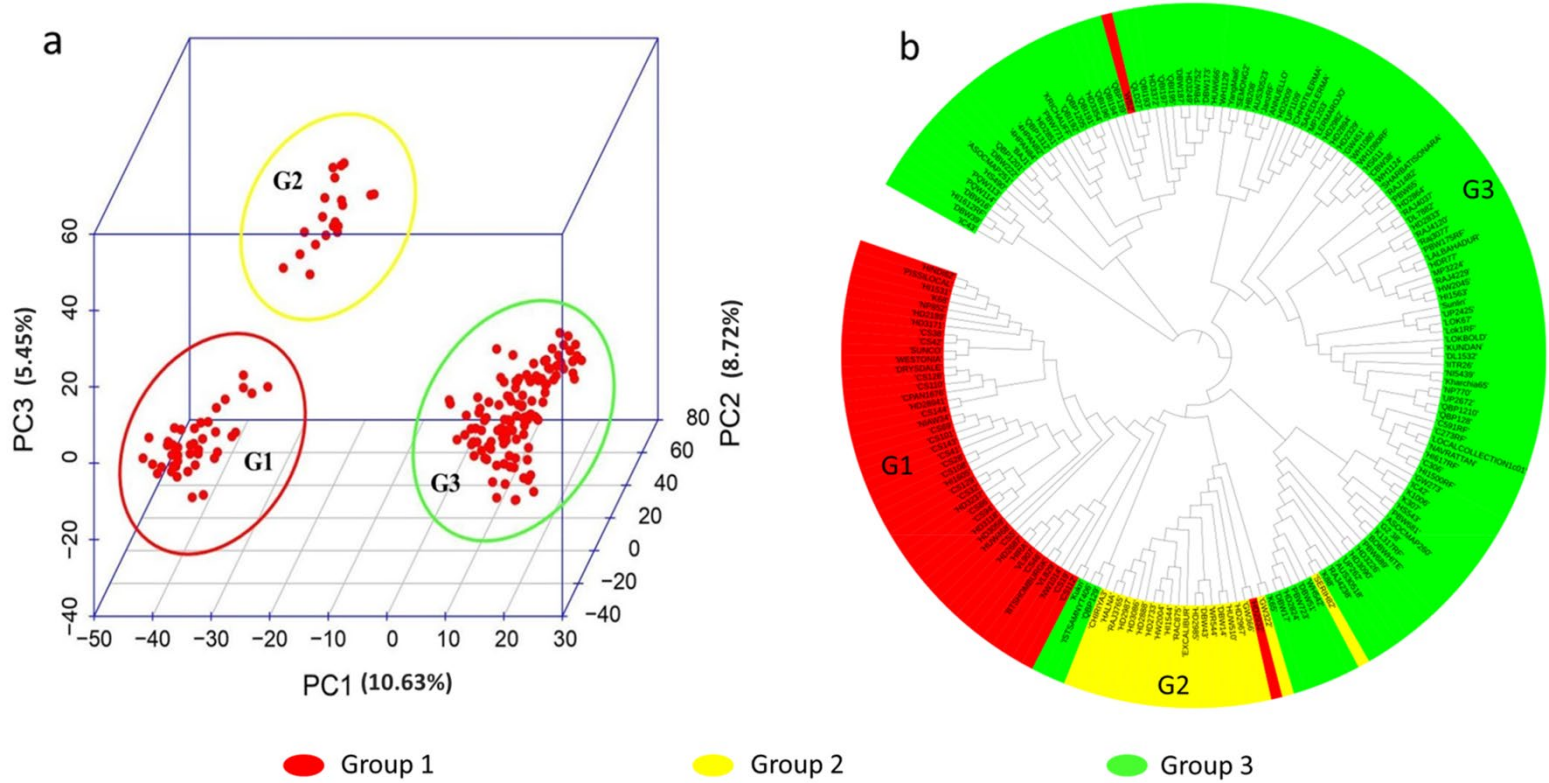
Three-dimensional principal component analysis plot (**a**), Neighbor-joining tree (**b**) inferring the population structure.

Population structure inferred by Principal Component Analysis (PCA) revealed three groups in the GWAS panel (Fig. 4a). The three groups consisted of 45 (G1), 20 (G2) and 119 (G3) genotypes respectively. The PC1, PC2 and PC3 accounted for 10.63%, 8.72% and 5.45% of the total variation respectively. The first three principal components were used as covariates in GWAS analysis to reduce the false positives. The Clustering methods (N-J tree) also revealed the three subpopulations, thus confirming the results of PCA (Fig. 4b). The G1 has most of the exotic lines, G2 constituted of some of the new Indian varieties and G3 was dominated by breeding lines. The Indian varieties were distributed in all three groups.

### Marker-trait associations

**Figure 5:**
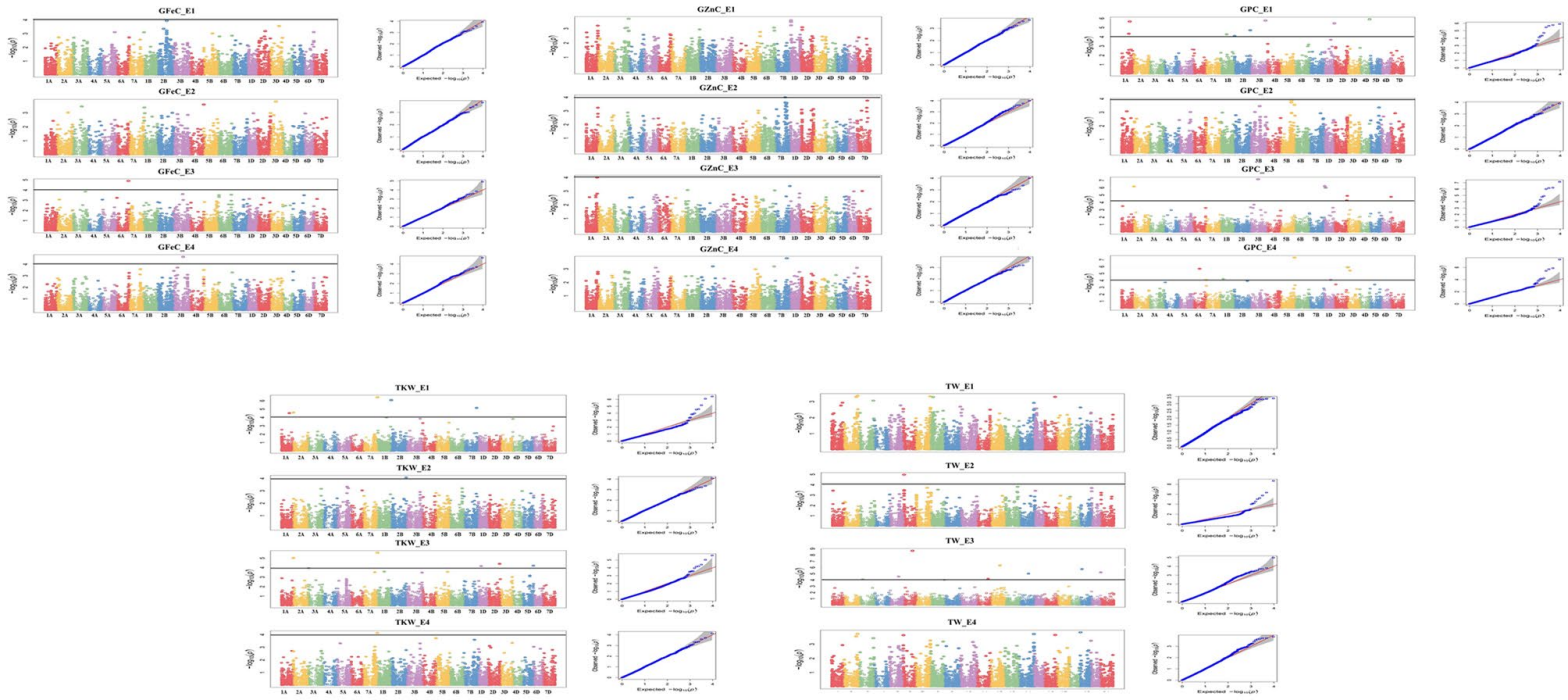
Manhattan and QQ plots for GFeC, GZnC, GPC, TKW and TW from IARI-New Delhi (E1), IARI-Indore (E2), GBPUAT-Pantnagar (E3), and across environments (E4).

A total of 55 MTAs were detected; 4 for GFeC, 2 for GZnC, 23 for GPC, 15 for TKW, and 11 for TW. The details of these MTAs are summarized in Table 5 and depicted as Manhattan plots in Fig. 5. The Q-Q plots illustrating observed associations between SNPs and grain micronutrient concentrations compared to expected associations after accounting for population structure are presented in Fig. 5.

**Table 5:** Marker trait associations (MTAs) detected for GFeC, GZnC, GPC, TKW, and TW in IARI-New Delhi (E1), IARI-Indore (E2), GBPUAT-Pantnagar (E3), and across environments (E4).

Environment	Marker	Chromosome	Position (bp)	*P* value	PV (%)
**Grain iron concentration (GFeC)**					
E1	*AX-94761251*	2B	458622067	0.0001	9.26
E3	*AX-94715803*	6A	585439024	1.25E−05	11.14
	*AX-95002032*	3A	637962272	0.0001	12.62
E4	*AX-94850629*	3B	473694803	2.26E−05	8.82
**Grain zinc concentration (GZnC)**					
E2	*AX-94422893*	7B	488410704	9.81E−05	7.60
E3	*AX-94651424*	1A	544723357	9.94E−05	6.35
**Grain protein content (GPC)**					
E1	*AX-95233137*	4D	482944373	1.23E−06	18.19
	*AX-94520117*	3B	745289825	1.70E−06	2.67
	*AX-94508066*	1A	398138141	2.16E−06	13.71
	*AX-95176802*	1D	492534106	3.42E−06	4.63
	*AX-94882514*	2B	790751851	1.94E−05	6.51
	*AX-95195514*	1A	354950241	4.57E−05	12.91
	*AX-95143788*	1B	302518719	5.34E−05	6.69
	*AX-94406025*	2B	9731087	7.80E−05	5.63
E2	*AX-95205856*	5B	536516629	0.0001	6.59
E3	*AX-94925607*	3B	377864897	7.14E−08	3.27
	*AX-95245523*	1D	10720570	6.06E−07	3.82
	*AX-95126447*	2A	24060452	6.93E−07	5.26
	*AX-94961243*	1D	58575112	9.85E−07	2.60
	*AX-94508535*	2D	640213277	1.27E−05	1.91
	*AX-94634646*	7D	6696023	1.71E−05	7.07
	*AX-94904784*	2D	620510699	5.02E−05	4.65
E4	*AX-94838908*	5B	689223068	5.04E−08	13.87
	*AX-94855510*	3D	22767467	1.33E−06	4.08
	*AX-95237023*	6A	307984014	2.00E−06	2.65
	*AX-95104691*	3D	130928915	3.72E−06	5.01
	*AX-94834634*	1B	137447851	6.01E−05	3.01
	*AX-94424536*	7A	21353471	7.77E−05	7.20
	*AX-94649651*	1D	315034709	7.77E−05	6.26
**Thousand Kernel weight (TKW)**					
E1	*AX-94939463*	7A	731882551	3.86E−07	12.07
	*AX-94680946*	2B	15684332	8.25E−07	10.83
	*AX-94471749*	7B	652928863	7.09E−06	4.84
	*AX-94691823*	2A	16066165	2.54E−05	3.71
	*AX-94466632*	1A	403028587	2.97E−05	1.82
	*AX-94871220*	1B	455211699	0.0001	2.10
	*AX-94665811*	3B	694325462	0.0001	9.49
	*AX-94936235*	4D	12599553	0.0001	7.48
E2	*AX-95082269*	2B	779856091	8.21E−05	1.17
E3	*AX-94939463*	7A	731882551	2.68E−06	10.13
	*AX-94442811*	2D	590677321	3.93E−05	3.72
	*AX-94731421*	5D	546679712	6.22E−05	2.90
	*AX-94857979*	1D	138208001	6.95E−05	5.27
	*AX-94406738*	3A	21223605	0.0001	5.60
E4	*AX-94939463*	7A	731882551	7.25E−05	8.09
**Test weight (TW)**					
E2	*AX-95222115*	6A	425861645	2.14E−09	7.98
	*AX-94480810*	5B	444085704	4.68E−07	2.05
	*AX-95008379*	5D	84317542	1.80E−06	7.39
	*AX-95076851*	6D	458362043	5.99E−06	7.65
	*AX-94916640*	7B	435815231	9.48E−06	7.23
	*AX-95071409*	5A	440264163	2.84E−05	2.98
	*AX-94623317*	4B	527009135	6.14E−05	2.20
	*AX-94795774*	3A	147285088	7.58E−05	1.65
	*AX-95166792*	1B	667825172	9.84E−05	2.81
E3	*AX-95197534*	6A	3851364	1.05E−05	13.79
E4	*AX-94519472*	5D	46312169	0.0001	4.21

### MTAs for GFeC

**Figure 6:**
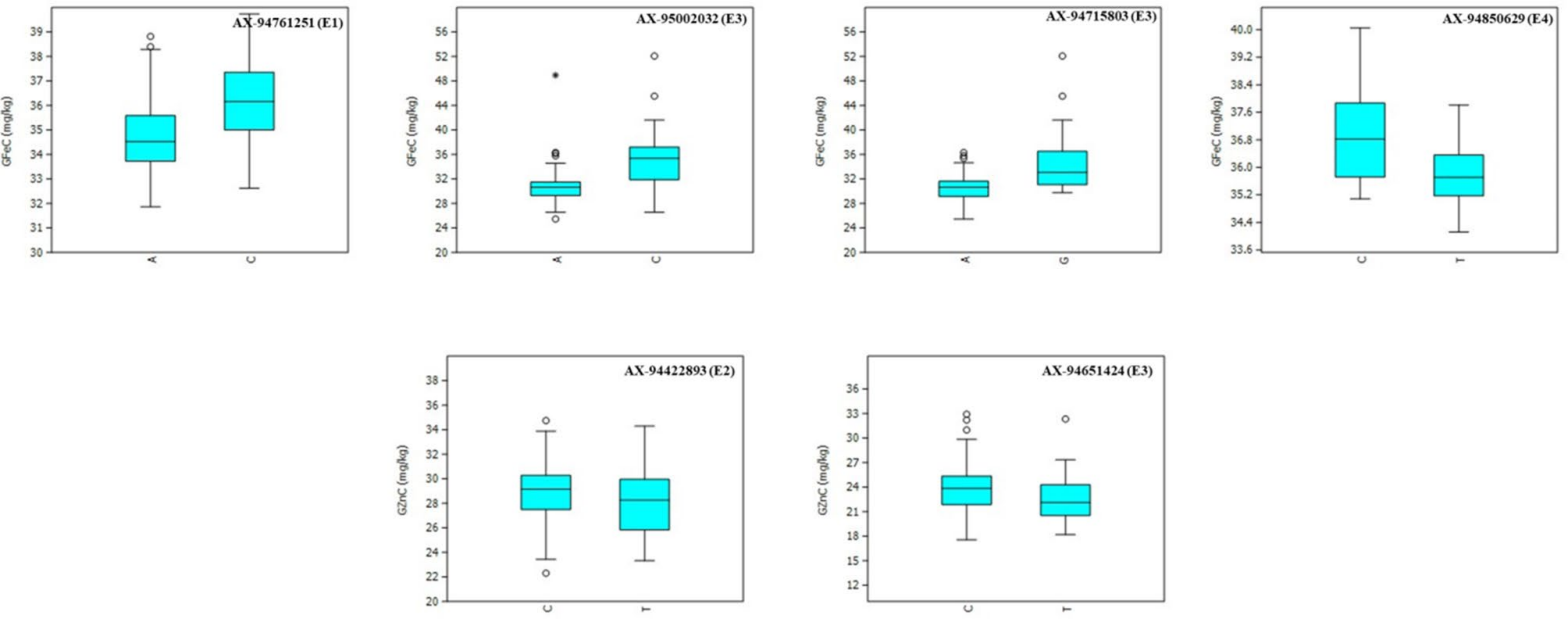
Allelic differences of the significant MTAs identified for GFeC and GZnC in IARI-New Delhi (E1), IARI-Indore (E2), GBPUAT-Pantnagar (E3), and across environments (E4).

A total of four significant MTAs were identified for GFeC in E1, E3, and E4 environments on chromosomes 2B, 3A, 3B, and 6A (Table 5; Fig. 5). The phenotypic variation (PV) explained by these SNPs ranged between 8.82 and 12.62%. A major SNP on chromosome 3A (*AX-95002032*) located at 637.9 Mb explained 12.62% of the PV, while another SNP on chromosome 6A (*AX-94715803*) located at 585.4 Mb explained 11.14% of PV, both were detected in E3. The other SNPs, *AX-94761251* on 2B and *AX-94850629* on 3B explained the PV of 9.26 and 8.82%, respectively. The SNP, *AX-95002032* had A and C alleles with a phenotypic average of 30.68 mg/kg and 36.14 mg/kg respectively. The SNP, *AX-94715803* had A and G alleles with a phenotypic average of 30.6 and 34.76 mg/kg respectively (Fig. 6).

### MTAs for GZnC

Two significant MTAs were identified for GZnC in E2 and E3 environments on chromosomes 1A and 7B (Table 5; Fig. 5). One SNP (*AX-94422893*) was identified on chromosome 7B, located at 488.4 Mb, and explained 7.60% of the PV, while another SNP on chromosome 1A (*AX-94651424*) located at 544.7 Mb explained 6.37% of PV. The SNP *AX-94422893* had C and T alleles with a phenotypic average of 29.03 and 28.13 mg/kg respectively. The SNP *AX-94651424* had C and T alleles with a phenotypic average of 24.04 and 22.51 mg/kg respectively (Fig. 6).

### MTAs for GPC

A total of 23 significant MTAs were identified for GPC across all environments and pooled mean, which were located on 13 different chromosomes viz., 1A, 1B, 1D, 2A, 2B, 2D, 3B, 3D, 4D, 5B, 6A, 7A, and 7D (Table 5; Fig. 5) and PV explained ranged from 1.91–18.19%. A major SNP detected in E1 on chromosome 4D (*AX-95233137*) located at 482.9 Mb explained 18.19% of the PV, while the SNPs on chromosome 1A, *AX-94508066* located at 398.1 Mb, *AX-95195514* located at 354.9 Mb explained 13.71% and 12.91% PV respectively. The SNP detected in E4 on chromosome 5B (*AX-94838908*) located at 689.2 Mb explained 13.87% of PV.

### MTAs for TKW

A total of 15 significant MTAs were identified for TKW across all the environments and pooled mean. The corresponding SNPs were assigned to 12 different chromosomes, namely, 1A, 1B, 1D, 2A, 2B, 2D, 3A, 3B, 4D, 5D, 7A, and 7B (Table 5; Fig. 5) and PV explained ranged from 1.17 to 12.07%. The SNP *AX-94939463* on chromosome 7A located at 731.8 Mb, was identified in the three environments namely, E1, E3, and E4, and explained the PV of 12.07, 10.13, and 8.09% respectively. This SNP had A and G alleles with a phenotypic average of 36.22 and 39.45 g, 32.86 and 37.95 g, 36.50 and 39.81 g at E1, E3, and E4 respectively. The other major SNPs identified were *AX-94680946* on chromosome 2B at 156.8 Mb explained a PV of 10.83% followed by *AX-94665811* on chromosome 3B at 694.3 Mb explained a PV of 9.49%. Both the SNPs were identified in E1.

### MTAs for TW

A total of 11 significant MTAs were identified for TW in E2, E3, and E4 on chromosomes 1B, 3A, 4B, 5A, 5B, 5D, 6A, 6D, and 7B (Table 5; Fig. 5). The SNPs explained 1.65–13.79% of PV. A major SNP detected at E3 on chromosome 6A (*AX-95197534*) located at 3.8 Mb explained 13.79% of the PV.

### Pleiotropic regions and stable MTAs

A stable MTA was identified for TKW on chromosome 7A in three environments i.e. E1, E3 and E4 (details are given in TKW paragraph of results section). The pleiotropic regions were identified for GPC and TKW on chromosome 1A (*AX-94508066&AX-94466632*), 2A (*AX-95126447&AX-94691823*) and 2B (*AX-94882514&AX-95082269*) between 398–403.1 Mb, 16–24.1 Mb and 777.8–790.8 Mb, respectively. They explained the considerable PV for GPC (13.71, 5.26, and 6.51%) and small PV for TKW (1.82, 3.71, and 1.17%).

### In silico analysis

In silico analysis identified 23 candidate genes associated with important MTAs of GFeC and GZnC (Table 6). The candidate genes identified for GFeC includes *TraesCS2B02G321500* (*Domain of unknown function DUF3475* and *Domain of unknown function DUF668*), *TraesCS3B02G295000 (Serine-threonine/tyrosine-protein kinase), TraesCS6A02G353900 (Zinc finger CCCH-type proteins) and TraesCS3A02G389000* (*F-box-like domain superfamily*) among others. The candidate genes for GZnC include *TraesCS7B02G266000 (Histone deacetylase domain superfamily* or *Ureohydrolase domain superfamily)* and*TraesCS1A02G365900 (SANT/Myb domain* or *Homeobox-like domain superfamily)* among others.

**Table 6:** Putative candidate genes identified for GZnC and GFeC.

Trait	SNP ID	Position (Mb)	Chr	TraesID	Putative candidate genes	Molecular function
GFeC	AX-94761251	458.62	2B	TraesCS2B02G321500	Domain of unknown function DUF3475 Domain of unknown function DUF668	Positive regulation of growth
				TraesCS2B02G321600	Zinc finger, GRF-type	Zinc ion binding
	AX-94850629	473.69	3B	TraesCS3B02G295000	Serine-threonine/tyrosine-protein kinase Wall-associated receptor kinase	Protein kinase activity Polysaccharide binding, ATP binding
				TraesCS3B02G294600	Rossmann-like alpha/beta/alpha sandwich fold Cytidyltransferase-like domain	Nicotinamide-nucleotide adenylyltransferase activity Catalytic activity ATPase
				TraesCS3B02G294700	Reticulon-like protein	Response to bacterium
				TraesCS3B02G294900	Glycoside hydrolase family 18 Serine-threonine/tyrosine-protein kinase	Hydrolase activity, Hydrolyzing O-glycosyl compounds, Protein kinase activity, ATP binding
				TraesCS3B02G294800	Bifunctional inhibitor/plant lipid transfer protein/seed storage helical domain	Lipid transport
				TraesCS3B02G295300	Wall-associated receptor kinase, C-terminal	Polysaccharide binding
	AX-94715803	585.43	6A	TraesCS6A02G353900	Zinc finger CCCH-type, G-patch domain	Nucleic acid binding, Metal ion binding
				TraesCS6A02G353700	Pheophorbide a oxygenase Rieske [2Fe-2S] iron-sulphur domain	Chlorophyllide a oxygenase [overall] activity, 2 iron, 2 sulfur cluster binding
				TraesCS6A02G353800	Serine incorporator/TMS membrane protein	Integral component of membrane
				TraesCS6A02G354100	Peptidase family M49, NUDIX hydrolase domain	Hydrolase activity
	AX-95002032	637.96	3A	TraesCS3A02G389000	F-box-like domain superfamily	Protein binding
				TraesCS3A02G389100	Spindle assembly checkpoint component Mad1	Mitotic spindle assembly checkpoint signaling
				TraesCS3A02G389200	Domain of unknown function DUF4094, Glycosyltransferase, family 31	Galactosyltransferase activity Hexosyltransferase activity
				TraesCS3A02G389400	Pentatricopeptide repeat	Protein binding
				TraesCS3A02G389300	Protein kinase-like domain superfamily	Protein kinase activity
GZnC	AX-94422893	488.41	7B	TraesCS7B02G266000	Histone deacetylase domain superfamily Ureohydrolase domain superfamily	Histone deacetylase activity Hydrolase activity
				TraesCS7B02G265800	Cytochrome P450	Heme binding Metal ion binding
				TraesCS7B02G265900	Myc-type, basic helix-loop-helix (bHLH) domain Helix-loop-helix DNA-binding domain superfamily	Protein dimerization activity
				TraesCS7B02G266100	Nucleotide-diphospho-sugar transferases	–
				TraesCS7B02G266200	PPM-type phosphatase domain	Phosphatase activity
	AX-94651424	544.72	1A	TraesCS1A02G365900	SANT/Myb domain, Homeobox-like domain superfamily	DNA binding
TKW	AX-94939463	731.88	7A	TraesCS7A02G560100	Polysaccharide biosynthesis domain superfamily	Xylan biosynthetic process
				TraesCS7A02G560000	Dehydrin	Response to abscisic acid Response to water Cold acclimation

## Discussion

The genetics of GZnC, GFeC, GPC, TKW, and TW has been studied using GWAS in the present study. The GWAS panel exhibited significant variability for all the investigated traits. The range of variability obtained in the present study for GZnC (17.56–56.93 mg/kg) and GFeC (25.47–52.09 mg/kg) is in the range reported previously^43–51^. The heritability estimates were higher (h^2^ ≥ 0.60) for the studied traits, however, moderate heritability was found in some environments eg., > 0.40 and < 0.60 for GFeC in E1 and E4, GZnC and GPC in E4 (Table 1). The comparable heritability estimates were also reported in previous studies for these traits^29,32,49–53^.

The two genotypes i.e. the Navrattan (landrace) and 2.38 (SHW) were among the best performing genotypes for GZnC (36.05 and 37.72 mg/kg), GFeC (37.87 and 38.27 mg/kg) and GPC (12.32% and 12.22%) based on the combined BLUPs across environments and hence can be efficiently utilized in breeding programmes. The genotype Lokbold was identified to be another good performer for GFeC (37.76 mg/kg), GZnC (35.26 mg/kg), and TKW (49.8 g) and therefore can also be given due consideration in breeding programmes (Table 2). The performance of many old Indian varieties was found to be better for GZnC, GFeC, and GPC than recently released cultivars and vice versa was the case for TKW and TW, confirming the dilution effect, i.e. increased efforts of plant breeders in enhancing grain yield led to an unintentional increase of more starchy endosperm, thus reductions in other important quality components in modern wheat varieties^54^.

The significant positive correlations among GZnC, GFeC, and GPC indicate the possibility of simultaneous improvement of these traits. This finding is in line with earlier reports^44,49–52^. Additionally, many studies suggested a common genetic basis for these traits through GWAS and conventional QTL studies^50,51,55,56^. Also, GFeC and GZnC showed either positive or no correlation with TKW and TW, indicating that the grain Zn and Fe can be increased without yield penalty. However, the study also shows the negative association of TW and TKW with GPC indicating yield penalty with the increase in GPC beyond a certain level^34^, thus it is suggested to improve protein quality profile with the optimum level of protein quantity required for the superior-quality end product.

The population structure inferred by PCA revealed three sub-populations in the GWAS panel (Fig. 4). Similarly, NJ-based clustering divided the whole set of 184 genotypes into three distinct clusters. The genotypes were clustered in the previous studies mainly based on pedigree, geographical, and evolutionary origin^29,31–33^. In the current study, G1 was dominated by exotic lines, G2 constituted new Indian varieties and G3 by breeding lines. Most significantly, the Indian varieties were mixed up with all the three groups, thus pointing towards their broad genetic base. The results also suggest that many new Indian varieties might have been bred by introgressing genes from exotic lines.

The LD decay over genetic or physical distance in a population determines the density of marker coverage needed to perform GWAS. A faster LD decay indicates that a higher marker density is required to capture the markers close enough to the causal loci^57^. In the present study, the LD decayed to its half from the maximum LD at 4.71 Mb for whole genome, 3.63 Mb for A, 5.63 Mb for B and 4.90 Mb for D sub-genomes (Fig. 3). A similar LD pattern of 5.98 Mb was reported in a set of Chinese wheat landraces^58^. In contrast, the whole genome LD decay was faster and it was at the distance of 2 Mb in a set of CIMMYT spring bread wheat lines^59^. In addition, the whole genome LD decay distance of 3 Mb was reported^60^. In contrast to faster LD decay, the slower LD decay distance of 22 Mb and 23 Mb respectively were found in a set of hexaploid wheat collections from Kazakhstan and in Mexican bread wheat landraces^61,62^. The variation in the LD pattern among different GWAS populations may be due to factors like selection, mutation, admixtures, non-random mating, etc.

A total of 55 MTAs were identified, 4 for GFeC, 2 for GZnC, 23 for GPC, 15 for TKW, and 11 for TW. The four MTAs were identified for GFeC on chromosomes 2B, 3A, 3B, and 6A and explained the PV ranging between 8.82 and 12.62% (Table 5; Fig. 5). Previously, MTAs for GFeC were reported on chromosome 3A^28^, on chromosome 3B^1,27^, further QTL were reported on 2B and 6A by^51,63^. The major SNP on chromosome 3A (*AX-95002032*) located at 637.96 Mb explained 12.62% of the PV, and the putative candidate gene linked with this marker is *TraesCS3A02G389000* (*F-box-like domain superfamily*). Interestingly, the E3 ubiquitin ligase complex containing the *FBXL5* (*F-box and leucine-rich repeats protein 5*) protein targets iron regulatory protein (*IRP2)*, the *FBXL5* accumulating under iron- and oxygen-replete conditions and degraded upon iron depletion^64,65^. These observations also hint at the possible role of *FBXL5* in iron sensing in plant systems. The SNP *AX-94715803* on chromosome 6A located at 585.43 Mb explained 11.14% of PV. The putative candidate gene linked with this marker is *TraesCS6A02G353900* (*Zinc finger CCCH-type, G-patch domain*). It is noteworthy that the zinc finger transcription factors, which control the functions of various genes, have a DNA binding domain that requires zinc or iron ions for its structural and functional stability and for activation^66^. The possible role of zinc finger protein in wheat grain zinc accumulation was also reported earlier^31,50,51^. The other two MTAs, *AX-94761251*, *AX-94850629* found on chromosome 2B at 458.62 Mb and on chromosome 3B at 473.69 Mb explained a respective PV of 9.26 and 8.82%, respectively. In silico analysis revealed the putative candidate genes *TraesCS2B02G321500* (Domain of unknown function *DUF3475*, *DUF668*) for *AX-94761251* and *TraesCS3B02G295000* (*Serine-threonine/tyrosine-protein kinase*) for *AX-94850629*. The possible role of protein kinases in grain iron and zinc accumulation in wheat was also indicated by other authors^1,27,28,50,51,67^. The protein kinases phosphorylate Fe and Zn proteins and are found to show greater interactions with Fe and Zn transporter proteins^68^, hence these are expected to have a potential role in grain micronutrients accumulation.

The two SNPs associated with GZnC found on chromosomes 1A and 7B with PV ranged from 6.35 to 7.60% (Table 5; Fig. 5). Previous studies also reported the QTLs on these chromosomes^28,31,33,51^. An SNP on chromosome 7B (*AX-94422893*) located at 488.41 Mb explained 7.60% of PV, this region encodes *TraesCS7B02G266000* (*Histone deacetylase domain superfamily*, *Ureohydrolase domain superfamily*). Histone deacetylases play an important role in gene regulation. The zinc ion acts as a cofactor and regulates the catalytic function of the classical HDAC family of enzymes (Class I, II, IV)^69^. Another SNP on chromosome 1A (*AX-94651424*) located at 544.72 Mb explained 6.37% of PV and codes for *TraesCS1A02G365900* (*SANT/Myb domain*, *Homeobox-like domain superfamily*). Interestingly, Arabidopsis *Myb* transcription factors positively regulate the biosynthesis of glucosinolates^70^ which in turn are involved in trade-off with Zn. This is evident from the studies on *Thlaspicaerulescens*, where Zn hyper accumulation decreased sinalbin (*p-hydroxybenzylglucosinolate*) concentration in shoots^71^.

Additionally, 23 MTAs identified for GPC, 15 for TKW, and 11 for TW. They explained the PV of up to 18.19%, 12.07%, and 13.79% respectively. A major SNP *AX-94939463* identified for TKW on chromosome 7A at 731.88 Mb, stably found in three environments namely, E1, E3, and E4, and explained the PV of 12.07, 10.13, and 8.09% in the respective environments. This SNP was found in the region codes for *TraesCS7A02G560100* (*Polysaccharide biosynthesis domain*). Interestingly, the polysaccharide synthesizing glycosyltransferases are the enzymes generally organized on Golgi bodies that catalyze the synthesis of more complex and highly branched polysaccharides^72^. Previously, two candidate genes namely, *TaSus1* and *TaGASR-A1* were reported on chromosome 7A^73^.

The positive and highly significant correlation between GFeC, GZnC and GPC (P < 0.001) in all the environments suggested the possibility of simultaneous improvement of these traits. The best performing lines like Navrattan, SHW 2.38, and Lokbold can be utilized as sources in the breeding pipeline and for developing mapping populations to discover QTLs for grain Zn and Fe concentration and protein content. Further, the promising SNPs on chromosome 1A, 7B for GZnC and 2B, 3A, 3B, and 6A for GFeC could be converted into breeder’s friendly Kompetitive Allele Specific PCR (KASP) markers to be used in marker-assisted selection (MAS) or targeted introgression to develop biofortified cultivars. The putative candidate genes identified need to be validated further to shed light on their functional role in grain Fe and Zn concentration.

### Ethical approval

The genotypes listed in the study were in wheat section of Division of Genetics, ICAR-IARI, New Delhi and all imported lines have been obtained through National Bureau of Plant Genetic Resources, New Delhi following the prescribed guidelines. Also, we have all the permissions and rights to collect and use the genotypes for research purpose. The experimental research and field experiments in the present study are duly approved by the institute research council and advisory committee of NDR.

## Supplementary Information

Supplementary Table S1.

Supplementary Table S2.
